# Hybrid Magnetorheological Composites for Electric and Magnetic Field Sensors and Transducers

**DOI:** 10.3390/nano10102060

**Published:** 2020-10-19

**Authors:** Ioan Bica, Eugen Mircea Anitas, Liviu Chirigiu

**Affiliations:** 1 West University of Timisoara, V. Parvan Avenue 4, 300223 Timisoara, Romania; ioan.bica@e-uvt.ro; 2 Joint Institute for Nuclear Research, 141980 Dubna, Russia; 3 Horia Hulubei, National Institute of Physics and Nuclear Engineering, 077125 Bucharest-Magurele, Romania; 4 University of Medicine and Pharmacy, 200396 Craiova, Romania; liviu_chirigiu@yahoo.com

**Keywords:** magnetorheological suspensions, magnetodielectric effects, electrical properties, carbonyl iron, iron oxide microfibers

## Abstract

We present a simple, low-cost, and environmental-friendly method for the fabrication of hybrid magnetorheological composites (hMCs) based on cotton fibers soaked with a mixture of silicone oil (SO), carbonyl iron (CI) microparticles, and iron oxide microfibers (μF). The obtained hMCs, with various ratios (Φ) of SO and μF, are used as dielectric materials for manufacturing electrical devices. The equivalent electrical capacitance and resistance are investigated in the presence of an external magnetic field, with flux density *B*. Based on the recorded data, we obtain the variation of the relative dielectric constant ($\epsilon_{r}{}^{\prime }$), and electrical conductivity (σ), with Φ, and *B*. We show that, by increasing Φ, the distance between CI magnetic dipoles increases, and this leads to significant changes in the behaviour of $\epsilon_{r}{}^{\prime }$ and σ in a magnetic field. The results are explained by developing a theoretical model that is based on the dipolar approximation. They indicate that the obtained hMCs can be used in the fabrication of magneto-active fibers for fabrication of electric/magnetic field sensors and transducers.

## 1. Introduction

Composite materials are a class of materials that consist of two or more distinct phases with significantly different physical or chemical properties, such that, when combined, lead to a new material with enhanced properties as compared to those of each individual phase [1]. While traditionally, the most composites consist of a continuous phase (e.g., metal or a polymer matrix) reinforced with a second phase in the form of a powder (e.g., fibers, particles, flakes), novel synthesis methods [2,3,4,5,6] allow a fine-tuning of the morphology of each phase, their relative proportion, distribution or crystallinity, thus greatly extending the range of possible applications.

Due to the ever increasing needs of technologies related to automobile, aerospace, or bio-medicine industries, in the last years the attention of scientific community has been largely focused on synthesis, characterization and application of smart composites, i.e., composites whose properties (mechanical, rheological, etc.) are largely influenced when exposed to an external magnetic field [7,8,9]. In particular, magnetorheological composites (MCs) have attracted an intense research interest, owing to their potential applications in a large number of multi-functional devices, including energy dissipation [10], vibration absorbing [11], muscle-like actuators [12], or sensors [13,14,15]. Recently, the application of MCs as sensors and transducers has become a hot research topic [16,17,18,19] due to their high socio-economic impact and the rapid development of various fabrication methods, including three-dimensional (3D) printing [20,21,22] or magnetorheological drawing lithography [23]. An excellent review in which some perspectives in the development of wearable polymer-based sensors are described has recently been published in Ref. [24].

Among the existing MCs, the hybrid ones comprising various types of microfibers (hMCs) show great potential, mainly since their internal structure that is induced by the yarns fibers prevents sedimentation of the magnetizable phase [25,26,27]. The hMCs are magnetic active materials and they consist of fibers that are made from natural polymers such as hemp, cotton, bamboo, and/or artificial polymers serving as a matrix, soaked with mixtures of solutions that are based on ferro/ferri-magnetic nano/micro-particles and additives.

Together with improved structural properties, both the matrix and the solution comprising the hMCs, usually also induce superior physical and chemical properties. Such features open up new opportunities for the application of hMCs in various fields. Promising candidates include protective polyester fabric with magnetic properties using a mixture of carbonyl iron and nano carbon black along with aluminium sputtering, which has been shown to have good microwave absorbing properties, particularly in the primary range of 8.2–12.4 GHz [28]. Good magnetic properties have been shown for staple yarns coated with barium ferrite, where the magnetizable phase leads to an increase in the saturation and residual induction [29]. When using cotton yarns that are covered with hard (barium hexaferrite), and soft (Black Toner 6745 CP-313) magnetic particles, it is shown that residual magnetism and coercive field intensity of the yarns are increasing with the magnetic powder content in the coating solution [30]. Smart textile fabrics obtained by using a coating method with NdFeB flake-like microparticles exhibit high magnetization required under special conditions [31]. In addition, when natural compounds are used, such as cotton fibers soaked with mixtures containing honey or turmeric powder, the magnetodielectric effects induced by a static magnetic field superimposed on a medium-frequency electric field, allows for hMCs to be used for the fabrication of medical devices [32].

However, in some applications in which the hMCs are required to function as sensors or transducers, a combined effect of different properties is required such as: good magnetic properties, low mass density, and preventing sedimentation processes. Here, we address this issue and manufacture hMCs that are based on cotton fibers, carbonyl iron (CI) microparticles, and in-house iron oxide microfibers (μF) recently obtained in Ref. [33]. The CI have a diameter of few microns, and μF have a diameter of few micrometers. The co-existence of two types of fillers with different chemical composition and average size, gives hMCs the bimodal character. The μF improve the magnetic properties of hMCs, have about 2.7 times less mass density as compared to the usual microparticles used in fabrication of hMCs [27,32,34,35], and, due to their fibrous structure, they also act as an anti-sedimentation medium. The obtained hMCs are used as dielectric materials in the fabrication of a plane capacitor. It is shown that a static magnetic field superimposed on a medium-frequency electric field, as well as the mass concentration of μF, sensibly influence the electrical conductivity and relative dielectric permittivity of hMCs.

## 2. Materials and Methods

**Figure 1:**
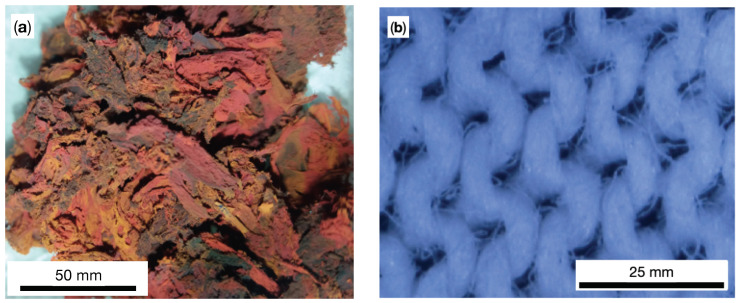
(Color online) (**a**) Image of μF. (**b**) Cotton fibers at 1000× magnification.

The raw materials used for fabrication of hMCs are:

- CI microparticles with an average size of 5μm and mass density $\rho_{\mathrm{CI}}=7.86$ g/cm${}^{3}$. The magnetization curve has been obtained in Ref. [36], where it has been shown that the remanent specific magnetization is 1.24 Am${}^{2}$/kg, the coercive field is 1.24 kA/m, and specific saturation magnetization of 210 Am${}^{2}$/kg, at a magnetic field intensity higher than 500 kA/m.
- SO with kinematic viscosity 100 cSt and mass density 0.97 g/cm${}^{3}$ at 298 K.
- μF (Figure 1a), with mass density of 2.875 g/cm${}^{3}$, prepared in a microwave plasma [33] from a mixture of CI, pentacarbonyl iron, and SO.
- Cotton fiber (GB) with a thickness of 0.42 mm. Its structure, visualized with an optical microscope, is shown in Figure 1b.

**Figure 2:**
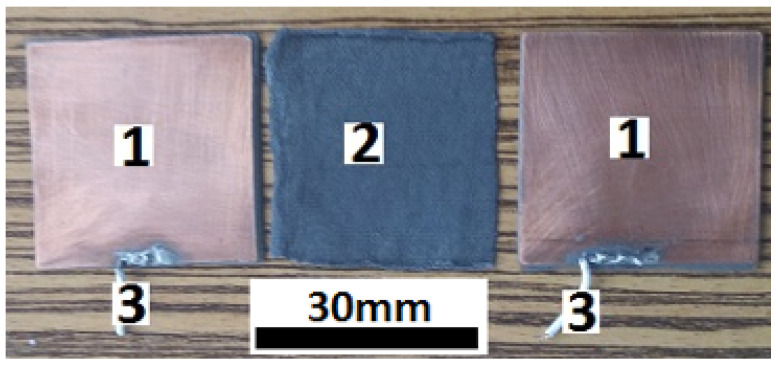
Textolite plates (view of the sides covered with copper)—position 1; hMC—position 2; and, Electrical connections—position 3.

**Figure 3:**
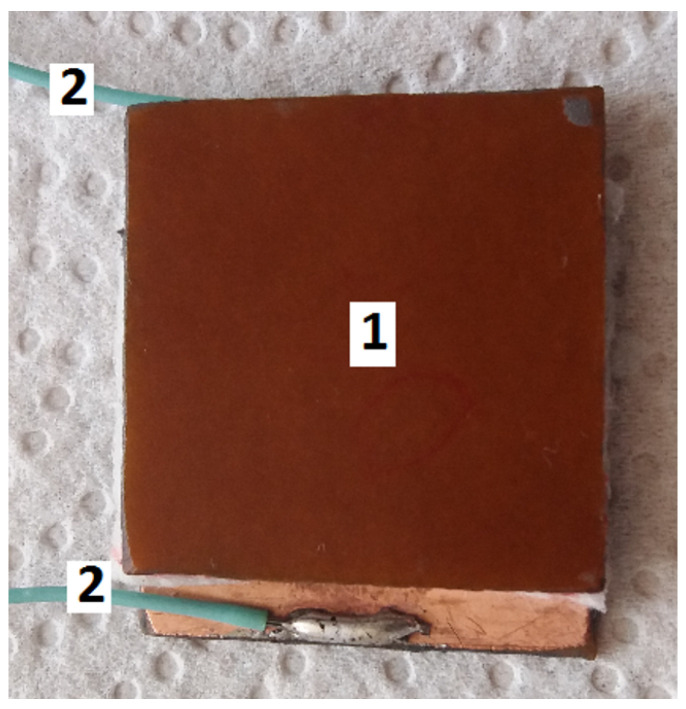
Electrical device (plane capacitor) with hMCs as dielectric material. Textolite plate (view of the side not covered with copper)—position 1; Electrical conductors—position 2.

The main steps in fabrication of hMCs are the following:

1. A quantity of 3.2 g of SO is mixed with 0.8 g of CI in a Berzelius glass beaker. The mixture is heated until the temperature reaches 423 K. At this temperature, the mixture is homogenized for 300 s, such that the humidity from CI is removed. At the end of this step, one obtains a mixture, denoted S${}_{1}$, in which the mass fraction of $\Phi_{\mathrm{CI}}=20\;\mathrm{wt}.\;\%$.
2. A quantity of 18 g consisting of CI (40 wt. %), μF (18 wt. %) and SO (wt. %) is also prepared in a Berzelius glass beaker placed on a heating source, and it is mixed for 300 s, after its temperature reaches 423 K. As such, the humidity from μF is also removed. The obtained sample is denoted by S.
3. After the sample S is brought to the room temperature, 9 g are extracted and deposited in a Berzelius glass beaker. Subsequently, to the sample S are added 9 g of SO. The solution is also mixed at 423 K for 300 s, and then is left to reach the room temperature. The obtained liquid solution is denoted by S${}_{4}$ and it consists of CI, μF and SO with mass fractions $\Phi_{\mathrm{CI}}=20$ wt. %, $\Phi_{\mu F}$ = 9 wt. %, and, respectively, $\Phi_{\mathrm{SO}}=71$ wt. %.
4. From the sample S${}_{4}$, one extracts 6 g of the liquid mixture, and pours it into a Berzelius glass beaker. A quantity of 1.2 g of CI and 10.8 g of SO is added, and the whole mixture is homogenized at 423 K for 300 s, and then is left to reach the room temperature. The obtained liquid solution is denoted by S${}_{3}$ and it consists of CI, μF and SO with mass fractions $\Phi_{\mathrm{CI}}=20$ wt. %, $\Phi_{\mu F}$ = 6 wt. %, and, respectively, $\Phi_{\mathrm{SO}}=74$ wt. %.
5. From the sample S${}_{3}$, one extracts 9 g of the liquid mixture, and pour it into a Berzelius glass beaker. A quantity of 1.8 g of CI and 7.2 g of SO is added, and the whole mixture is homogenized at 423 K for 300 s, and then it is left to reach the room temperature. The obtained liquid solution is denoted by S${}_{2}$ and it consists of CI, μF and SO with mass fractions $\Phi_{\mathrm{CI}}=20$ wt. %, $\Phi_{\mu F}$ = 3 wt. %, and, respectively, $\Phi_{\mathrm{SO}}=77$ wt. %. Table 1 summarizes the composition of samples obtained in these steps.
6. One cuts eight plates of textolites in the form of squares with edge length 30 mm. One side of each plate is covered with copper, as shown at position 1 in Figure 2. Additionally, four pieces of GB are prepared with the same dimensions. Each piece has a mass of $m_{\mathrm{GB}}=0.125$ g.
7. The GB obtained at the previous step is soaked, by turn, with a volume of 0.8 cm${}^{3}$ of the solutions listed in Table 1. As such one obtains the bimodal hMCs with the mass fractions listed in Table 2.
8. Each hMC is placed between the copper sides of two textolite plates. Thus, one obtains the the electrical device, as shown in Figure 3.

## 3. Structural and Magnetic Characterization

**Figure 4:**
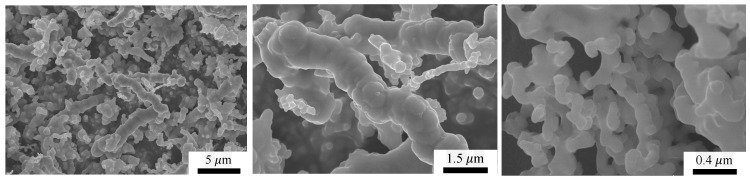
SEM images of μF at increasing magnifications from **left** to **right**.

**Figure 5:**
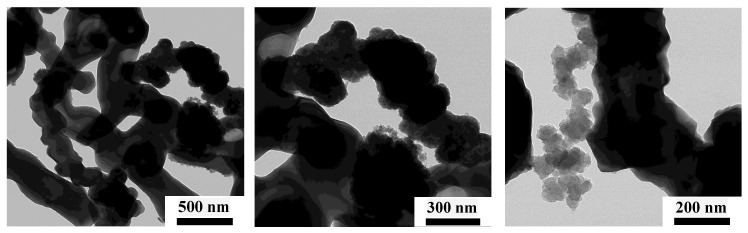
TEM images of μF at increasing magnifications from **left** to **right**.

**Figure 6:**
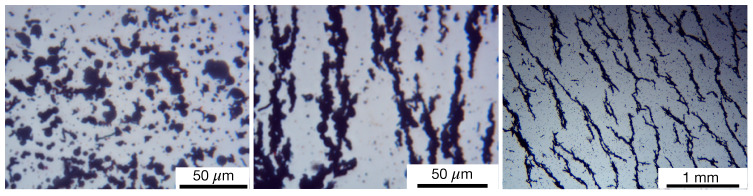
(Color online) Optical microscopy of: (**Left**) CI (black disks) and μF (black rod-like structures) without an external magnetic field; (**Middle**) CI (black disks) and μF (black rod-like particles) with an external magnetic field. Magnetic field is in the N-S direction; and, (**Right**) chain-like aggregates of μF in an external magnetic field. Magnetic field is in the NW-SE direction. The magnetic flux density is about 25 mT.

Scanning electron microscopy (SEM), transmission electron microscopy (TEM), and optical microscopy (OM) were used to characterize the surface morphology of the fibers. In the case of SEM, was used a catalyst powder supported on carbon tape, and the results indicate that the microfibers form a complex hierarchical network of multifractal type, as shown in Figure 4 [33]. SEM and TEM (Figure 5) show that the diameters of the fibers are between 0.25 μm and 2.20 μm, while energy dispersive X-ray spectroscopy of the microfibers indicates that thy consist of iron oxides [33]. OM shows that, in the absence of a magnetic field, the system of CI with μF form random aggregates (Figure 6 left part), while in the presence of an external magnetic field, the microfibers with or without CI form chain-like structure oriented along the magnetic field lines (see Figure 6 middle part, right part). The identification of crystallographic phase of μF was performed by X-ray diffraction, with a DMAX-2500, Rigaku (Japan) diffractometer, and the results confirm the presence of iron oxides [33].

For the analysis of magnetic properties, 276 g of μF is introduced in the measuring cell of a magnetometer and the measurements are performed under sine waveform driving field conditions by means of a laboratory-made ac induction hysteresis graph that is described in Ref. [37]. The saturation specific magnetization is found to be 22.7 Am${}^{2}$/kg, and is obtained at 477 kA/m, the remanent specific magnetization is 2.86 Am${}^{2}$/kg, and the intensity of coercive magnetic field is 12.33 kA/m [33]. Similarly, for CI microparticles, it has been found that the saturation specific magnetization is about 251.3 Am${}^{2}$/kg [33].

## 4. Results and Discussion

**Figure 7:**
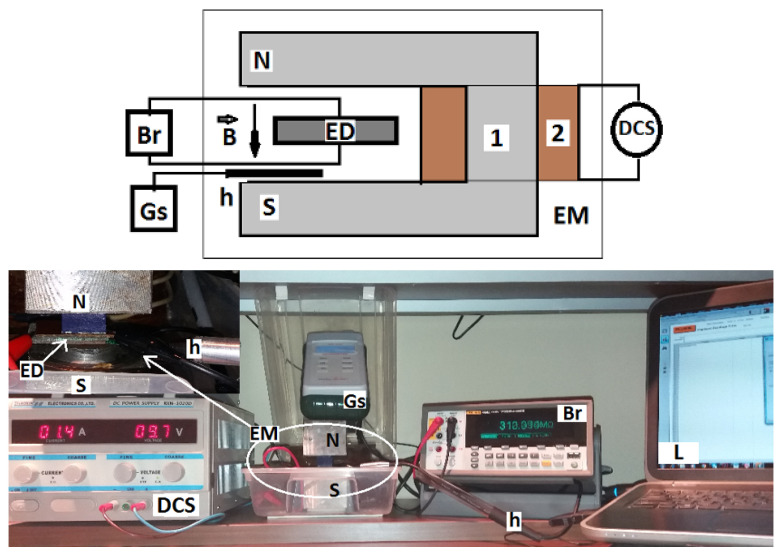
(Upper part) Overall configuration of the experimental setup. 1—magnetic yoke; 2—coil; EM—electromagnet; N and S—magnetic poles; B—magnetic flux density vector; DCS—continuous source current; ED—electrical device; Gs—Gaussmeter; h—Hall probe; and, Br—impedance-meter. (Lower part) Photo of the experimental setup. L—computing unit.

The experimental setup used for investigating the influence of the magnetic field and of μF on the electrical conductivity and on the relative dielectric permittivity is described in Figure 7 (upper part). A photo is presented in Figure 7 (lower part), and it shows that the setup consists from an electromagnet (EM), a source of continuous current (DCS), a Gaussmeter (Gs) with a Hall probe (h), an impedance meter (Br), and a computing unit (L).

The electrical device ED and the Hall probe h of the Gaussmeter Gs are fixed between the N and S poles of the electromagnet. The terminals of ED are connected to the impedance-meter. For fixed values of the magnetic flux densities B, one measures, with an accuracy of ±10%, the electrical and parallel components of the equivalent electrical circuit at time intervals of 10 s, after the magnetic field is applied. The electrical voltage at the terminals of the impedance meter is fixed at 1 V and the impedance at 10 kΩ.

### 4.1. Measurements of Electrical Capacitance and Resistance

**Figure 8:**
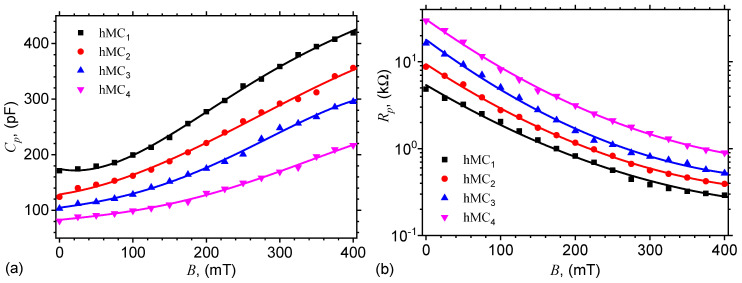
Equivalent electrical capacitance $C_{p}$ (**a**) and resistance $R_{p}$ (**b**) of hMCs, as a function of magnetic flux density *B*.

**Figure 9:**
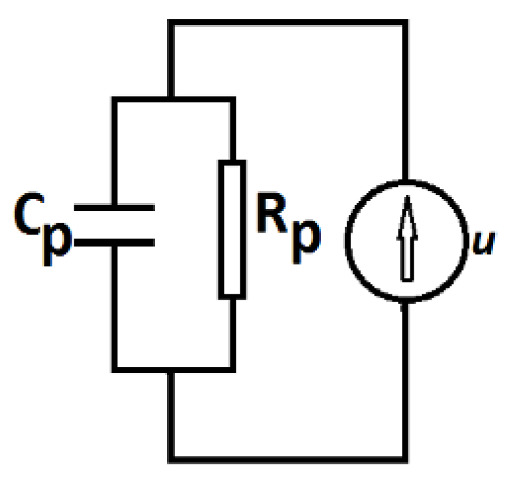
The equivalent electrical circuit of ED. $C_{p}$—equivalent electrical capacitance; $R_{p}$—equivalent electrical resistance; *u*—electrical voltage generated by the internal source of the impedance meter.

The capacitance $C_{p}$ and resistance $R_{p}$ of the plane capacitor are measured at the frequency of 1 MHz. The values of the magnetic flux densities are between 0 and 450 mT, and are recorded in steps of 25 mT. Figure 8 shows the obtained results. The results show that, for a fixed mass fraction of μF, the capacitance increases with flux density, while the resistance decreases. However, at a fixed value of flux density, the capacitance decreases with increasing mass fraction of μF, while the resistance increases. The obtained results suggest that the equivalent electrical scheme of the electrical device can be represented as in Figure 9.

**Figure 10:**
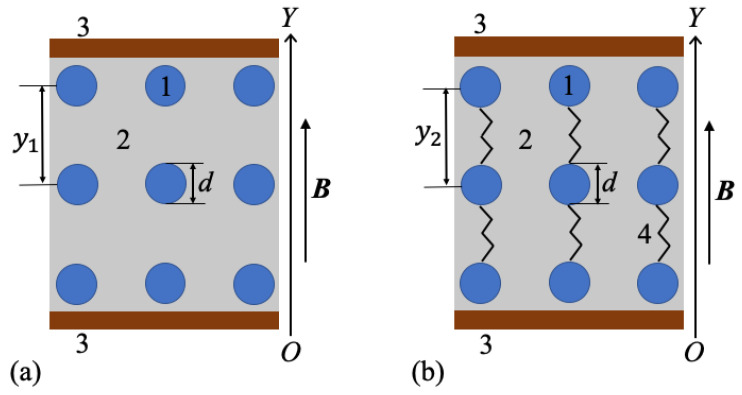
Model of magnetic dipoles in hMCs. (**a**): without μF. (**b**): with μF. 1—magnetic dipoles; 2—GB with SO; copper electrode; 4—μF; *d*—average diameter of a magnetic dipole; $y_{1}$ and $y_{2}$—distances between center-of-masses of two neighboring magnetic dipoles; B—magnetic flux density vector; OY—coordinate axis.

When a magnetic field with flux density of about 25 mT is applied, CI form chain-like aggregates. The equivalent schematic structure is shown in Figure 10.

### 4.2. Theoretical Model and Comparison with Experimental Data

When a magnetic field is applied (t=0), the CI microparticles become magnetic dipoles that are arranged in chain-like aggregates. The magnetic moment of each dipole from hMCs can be calculated according to [38]:

(1) $$m=0.5\pi d^{3}\frac{B}{\mu_{0}},$$

where *d* is the average diameter of the magnetic dipole, B is the magnetic flux density, and $\mu_{0}=4\pi \times 10^{-7}$ H/m is the vacuum permeability. In a magnetic field, the magnetic dipole chains arrange along the direction of B. Magnetic interactions occur between the magnetic dipoles from each chain. The OY projection of the corresponding intensity can be written as [38]:

(2) $$F_{\mathrm{my}}=-\frac{3\mu_{s}\mu_{0}m^{2}}{\pi y^{4}},$$

where $\mu_{s}≃1$ is the relative magnetic permeability of SO, *m* is the dipolar magnetic moment, and *y* is the distance between the mass centers of the dipoles.

Along the OY axis, is installed a resistive force of type $F_{\mathrm{ry}}=ky$ between the yarns of the cotton fibers, μF and magnetic dipoles, and that is opposed to the force $F_{\mathrm{my}}$. Thus, by taking into account Equations (1) and (2), at equilibrium, one can write [38]:

(3) $$k\frac{\partial y}{\partial t}+\frac{3\pi }{4\mu_{0}}\frac{d^{6}B^{2}}{y^{4}}=0.$$

By using the conditions y=δ at t=0, and y=y at t≠0, where $\delta =d\Phi_{\mathrm{CI}}^{-1/3}$ [38] is the initial distance between the mass centers of the dipoles, Equation (3) becomes:

(4) $$y=\delta {\left(1-\frac{15\pi }{4\mu_{0}}\frac{d^{6}B^{2}}{k\delta^{5}}t\right)}^{1/5}.$$

For a given mass fraction $\Phi_{\mathrm{CI}}$ of CI, the corresponding volume fraction can be written as $\Phi_{\mathrm{CI}}^{\mathrm{vol}}=\rho_{\mathrm{SO}}\Phi_{\mathrm{CI}}/\rho_{\mathrm{CI}}$. Here $\rho_{\mathrm{SO}}=0.97$ g/cm${}^{3}$ and $\rho_{\mathrm{CI}}=7.86$ g/cm${}^{3}$ are the densities of SO and, respectively, of CI. Thus, for numerical value d=5μm, one obtains δ=17.17μm. In the case of hMCs with CI and μF, one can write similarly that $\delta_{\mathrm{CI},\mu F}=d\Phi_{\mathrm{CI}}^{-1/3}{\left(1+\Phi_{\mu F}\right)}^{1/3}$. For calculating the the volume fraction of $\Phi_{\mu F}^{vol}$, one use a similar relation as in the case of CI microparticles, i.e., $\Phi_{\mu F}^{\mathrm{vol}}=\rho_{\mathrm{SO}}\Phi_{\mu F}/\rho_{\mu F}$. Because the density of microfibers is $\rho_{\mu F}=2.875$ g/cm${}^{3}$, then for numerical values of mass fractions $\Phi_{\mathrm{CI}}$ and μF from Table 2, one obtains the distances between the mass centers of magnetic dipoles in the system CI with μF as: $\delta_{\mathrm{CI},\mu F}=18.29\;\mu$m for hMC${}_{1}$, $\delta_{\mathrm{CI},\mu F}=18.35\;\mu$m for hMC${}_{2}$, $\delta_{\mathrm{CI},\mu F}=18.40\;\mu$m for hMC${}_{3}$, and $\delta_{\mathrm{CI},\mu F}=18.45\;\mu$m for hMC${}_{4}$. These results show that $\delta_{\mathrm{CI},\mu F}$ increases with increasing the quantity of μF. As a result, at B=0, the capacitance $C_{p}$ decreases, and the resistance increases $R_{p}$, as experimentally confirmed in Figure 10.

In the simplified model that is shown in Figure 10, one can consider that each pair of neighbouring magnetic dipoles, situated at distance *y* from each other, form a plane microcapacitor and, respectively, a linear microresistor. Their capacitance and resistance can be approximated by:

(5) $$C_{y}=\frac{\epsilon_{0}\epsilon_{0r}^{{}^{\prime }}\pi d^{2}}{4y},$$

and respectively by

(6) $$R_{y}=\frac{4y}{\sigma_{0}\pi d^{2}},$$

where $\epsilon_{0}$ is the vacuum dielectric constant, $\epsilon_{0r}^{{}^{\prime }}$ is the relative dielectric permittivity at B=0, and $\sigma_{0}$ is the electrical conductivity at B=0.

By denoting with *h* the thickness of the hMCs, then the maximum number of dipoles in the chain $n_{1}$ can be approximated by $n_{1}=h/d$. Because, within the dipole chains, the microcapacitors are arranged in series, the n for $n_{1}\gg 1$, the electrical capacitance of a single chain can be written by:

(7) $$C_{1}\equiv \frac{C_{x}}{n_{1}-1}=\frac{\pi \epsilon_{0}\epsilon_{0r}^{{}^{\prime }}d^{3}}{4hy}.$$

Similarly, the microresistors are also arranged in series and, for $n_{1}\gg 1$, the electrical resistance of a single dipole chain can be approximated by:

(8) $$R_{1}\equiv n_{1}R_{x}=\frac{4hy}{\sigma_{0}\pi d^{3}}.$$

The total number of dipoles inside hMCs is determined by the volume fraction $\Phi_{\mathrm{CI}}$ of microparticles, which is:

(9) $$N\equiv \frac{\Phi_{\mathrm{CI}}V}{V_{d}}=\frac{6\rho_{\mathrm{SO}}\Phi_{\mathrm{CI}}Llh}{\pi \rho_{\mathrm{CI}}d^{3}},$$

where *V* is the volume of GB, $V_{d}$ is the volume of a single magnetic dipole, and L,l and *h* are the length, width, and respectively, the thickness of hMCs.

The total number of chains within hMCs can be approximated by:

(10) $$n_{2}\equiv \frac{n}{n_{1}}=\frac{6\rho_{\mathrm{SO}}\Phi_{\mathrm{CI}}Llh}{\pi \rho_{\mathrm{CI}}d^{2}}.$$

Subsequently, since the total capacitance and resistance are given by $C_{p}=n_{2}C_{1}$, and respectively by $R_{p}=R_{1}/n_{2}$, by using Equations (7), (8) and (10), one obtains:

(11) $$C_{p}=C_{0p}{\left(1-\frac{15\pi }{4\mu_{0}}\frac{d^{6}B^{2}}{k\delta^{5}t}\right)}^{-1/5},$$

and respectively:

(12) $$R_{p}=R_{0p}{\left(1-\frac{15\pi }{4\mu_{0}}\frac{d^{6}B^{2}}{k\delta^{5}}t\right)}^{1/5},$$

In the above equations, *y* is given by Equation (4), $C_{0p}=1.5\epsilon_{0}\epsilon_{0r}^{{}^{\prime }}\rho_{\mathrm{SO}}\Phi_{\mathrm{CI}}Lld{\left(h\delta \rho_{\mathrm{CI}}\right)}^{-1}$ is the electrical resistance of ED at B=0, $R_{0p}=h\delta \rho_{\mathrm{CI}}{\left(1.5\sigma_{0}\rho_{\mathrm{SO}}\Phi_{\mathrm{CI}}Lld\right)}^{-1}$ is the electrical resistance of ED at B=0, and *k* is the particle friction coefficient.

By neglecting the edge effects, the equivalent electrical capacitance and electrical resistance can be also expressed as: $C_{p}=\epsilon_{0}\epsilon_{r}^{{}^{\prime }}Llh^{-1}$, and respectively $R_{p}=h{\left(\sigma Ll\right)}^{-1}$, where $\epsilon_{r}^{{}^{\prime }}$ and σ are the relative dielectric permittivity, and, respectively, the electrical conductivity of hMCs at B≠0. For numerical values L=l=30 mm, h=0.42 mm, and $\epsilon_{0}=8.85\;$pF/m, one obtains the relative dielectric permittivity of hMCs, i.e.,:

(13) $$10^{3}\epsilon_{r}^{{}^{\prime }}≃53\times C_{p}\left(\mathrm{pF}\right).$$

**Figure 11:**
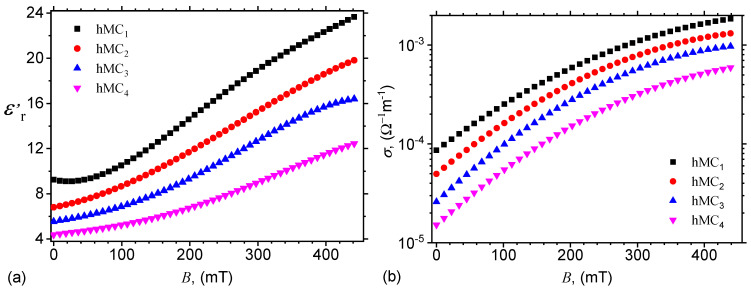
Relative dielectric permittivity $\epsilon_{r}^{{}^{\prime }}$ (**a**) and electrical conductivity σ (**b**), as a function of magnetic flux density *B*.

Subsequently, by introducing in the above equation the functions $C_{p}=C_{p}{\left(B\right)}_{\mathrm{hMCs}}$ from Figure 8a, one obtains the variation of relative dielectric permittivity with magnetic flux density *B* as shown in Figure 11a. The results show that for a fixed value of the quantity of μF, the relative dielectric permittivity $\epsilon_{r}^{{}^{\prime }}$ increases with magnetic flux density *B*, while for a fixed value of *B*, $\epsilon_{r}^{{}^{\prime }}$ decreases with increasing the quantity of μF. By introducing Equation (11) in Equation (13), one can see that the relative dielectric permittivity $\epsilon_{r}^{{}^{\prime }}$ also increases with magnetic flux density and decrease with the quantity of microfibers, thus supporting the qualitative behavior of experimental data in Figure 11.

The electrical conductivity of hMCs is obtained from the expression above, when the edge effects are neglected and, for the same numerical values of L,l and *h*, it is given by:

(14) $$10^{4}\times \sigma \left(\Omega^{-1}m^{-1}\right)≃\frac{1.667}{R_{p}\left(k\Omega \right)}.$$

Subsequently, by introducing the variation of $R_{p}$ from Figure 8b in the above equation, one obtains the variation of electrical conductivity with magnetic flux density *B*, as illustrated in Figure 11b. The results show that, at a fixed value of magnetic flux density, an increase of the quantity of μF leads to a decrease of the electrical conductivity of hMCs. This effect arise due to the increase of $R_{0p}$ with increasing the distance between the mass centers of the dipoles. This is in agreement with the theoretical expression of $R_{0p}$ (after Equation (12)). However, for a fixed value of the quantity of μF, the electrical resistance decreases with increasing *B*. This is the result of the increase of the magnetic field interaction, and it leads to a sensible change of the electrical conductivity in hMCs when the magnetic flux density is varied.

In order to determine the structural viscosity of the hMCs, one extracts the particle friction coefficient from Equation (12) and obtains:

(15) $$k=4.6875\times {\left(\frac{d}{\delta_{\mathrm{CI},¯F}}\right)}^{5}\frac{10^{-4}B^{2}\left(\mathrm{mT}\right)}{\left(1-{\left(\frac{R_{p}}{R_{0p}}\right)}^{5}\right)}.$$

**Figure 12:**
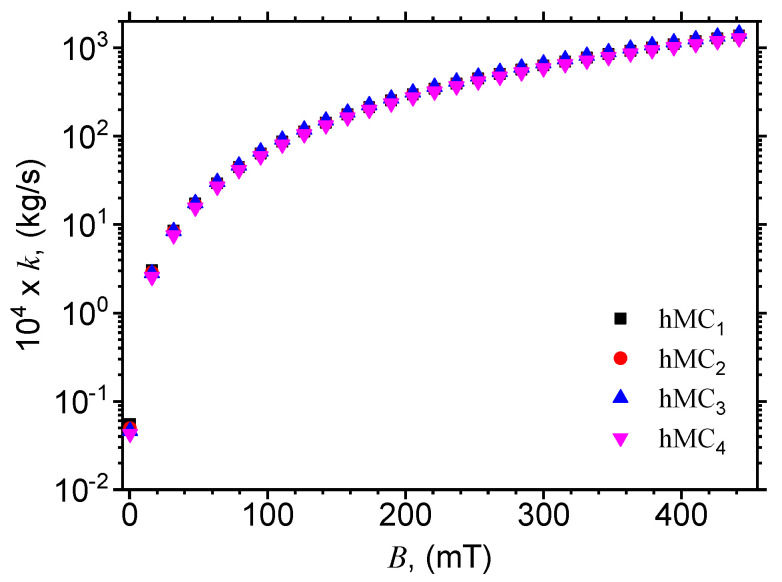
Variation of the particle friction coefficient *k* of the particles inside hMCs, with the magnetic flux density *B*.

**Figure 13:**
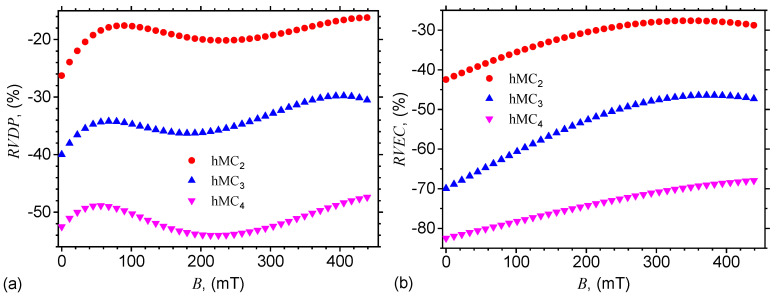
Relative variation of dielectric permittivity (**a**), and of electrical conductivity (**b**) of hMCs with CI and μF with magnetic flux density.

By using numerical values d=5μm and the variation of resistance $R_{p}$ from Figure 8b, one obtains the variation of the particle friction coefficient *k* as a function of the magnetic flux density, as shown in Figure 12. Note that the particle friction coefficient *k* is related to the structural viscosity (see Ref. [38]), which, in turn, is sensibly influenced by magnetic flux density *B*. This behavior is reflected also in Figure 13, if one consider that the magnetic properties of CI microparticles are much higher as compared to those of μF, as mentioned in Section 2.

The contribution of μF to the relative dielectric permittivity and, respectively, to the electrical conductivity of hMCs, can be quantitatively described by the following expressions:

(16) $$RVDP\left(\%\right)=\left(\frac{\epsilon_{\mathrm{CI},¯F}}{\epsilon_{\mathrm{CI}}}-1\right)\times 100,$$

and respectively by:

(17) $$RVEC\left(\%\right)=\left(\frac{\sigma_{\mathrm{CI},¯F}}{\sigma_{\mathrm{CI}}}-1\right)\times 100.$$

In the above equations, $\epsilon_{\mathrm{CI},¯F}$ is the relative dielectric permittivity of hMCs with CI and μF, $\epsilon_{\mathrm{CI}}$ is the relative dielectric permittivity of hMCs with CI, $\sigma_{\mathrm{CI},¯F}$ is the electrical conductivity of hMCs with CI, and μF, and $\sigma_{\mathrm{CI}}$ is the electrical conductivity of hMCs with CI.

By introducing the variation of dielectric permittivity from Figure 11a in Equation (16), one obtains the relative variation of dielectric permittivity with magnetic flux density for hMCs with CI and μF, as presented in Figure 13a. The results show the presence of maxima and minima, which arise due to the variation of structural viscosity of hMCs with a magnetic field [32].

Finally, by using the variation of electrical conductivity from Figure 11b with magnetic flux density *B*, in Equation (17) one obtains the relative variation of electrical conductivity with *B* for hMCs with CI and μF. The results are presented in Figure 13b and they show that, for a fixed value of *B*, the relative electrical conductivity increases, in absolute value, due to the increase of the quantity of μF. However, when the quantity of microfibers is fixed, the increase of relative electrical conductivity in a magnetic field arises due to the increase of intensities of magnetic interactions from hMCs with increasing *B* and as a consequence of an increase of the electrical conductivity.

Note that, in Figure 13b, hMC2 and hMC3 have maxima at B≃350 mT. At these values of *B*, the iron microfibers begin to interact, and they are attracted to the space between the magnetic dipoles. Therefore, the distance y (see Equation (4)) between dipoles slightly increase, which leads to a slight decrease of the electrical conductivity of hMC2 and hMC3, and thus to a decrease of RVEC.

## 5. Conclusions

In this work, we fabricate hMCs that are based on cotton fibers soaked with silicone carbonyl iron microparticles and various concentrations of iron oxide microfibers. The obtained hMCs are used as dielectric materials for fabrication of electrical devices in the form of plane capacitors. The corresponding equivalent electrical capacitance and resistance are measured in a static magnetic field with flux densities up to 450 mT, superimposed on a medium-frequency alternating electric field.

The recorded measurements are used to show that, for a fixed value of the magnetic flux density, both the relative dielectric permittivity $\epsilon_{r}^{{}^{\prime }}$ and electrical conductivity σ sensibly decrease with increasing the quantity of microfibers. It is also shown that, for a fixed value of the quantity of microfibers, $\epsilon_{r}^{{}^{\prime }}$ and σ increase with magnetic flux density. A theoretical model that explains the observed effects is developed in the framework of dipolar approximations.

The possibility to control the electrical properties and magnetodielectric effects by tuning either an external magnetic field or the quantity of microfibers makes the obtained hMCs very good candidates in the fabrication of magneto-active fabrics useful for sensors and transducers of electric and/or magnetic fields.

## Figures and Tables

**Table 1:** The mass fractions $\Phi_{\mathrm{CI}}$, $\Phi_{\mathrm{SO}}$, and $\Phi_{\mu F}$ of CI, SO, and respectively of μF inside the liquid solutions $S_{i}$, i=1,2,3,4.

Sample	$\Phi_{\mathrm{CI}}$ (wt. %)	$\Phi_{\mathrm{SO}}$ (wt. %)	$\Phi_{\mu F}$ (wt. %)
$S_{1}$	20	80	0
$S_{2}$	20	77	3
$S_{3}$	20	74	6
$S_{4}$	20	71	9

**Table 2:** The mass fractions $\Phi_{\mathrm{GB}}$, $\Phi_{\mathrm{CI}}$, $\Phi_{\mathrm{SO}}$, and $\Phi_{\mu F}$ of GB, CI, SO, and, respectively, of μF composing the hMC${}_{i}$, *i* = 1, 2, 3, 4.

Sample	$\Phi_{\mathrm{GB}}$ (wt. %)	$\Phi_{\mathrm{CI}}$ (wt. %)	$\Phi_{\mathrm{SO}}$ (wt. %)	$\Phi_{\mu F}$ (wt. %)
$hMC_{1}$	17.24	16.55	66.21	0.00
$hMC_{2}$	17.24	16.55	63.73	2.48
$hMC_{3}$	17.24	16.55	61.25	4.96
$hMC_{4}$	17.24	16.55	58.77	7.44

## Data Availability

The raw data required to reproduce these findings are available to download from https://dx.doi.org/10.6084/m9.figshare.12058944. The processed data required to reproduce these findings are available to download from https://dx.doi.org/10.6084/m9.figshare.12058944.
